# Sources of Comfort

**Published:** 1868-05

**Authors:** 


### SOURCES OF COMFORT.

In all of life's troubles she had three sources of comfort, prayer, the house of God, and the reading of the Scriptures. During the "July Riots," when an infuriated crowd blocked the streets to plunder and then burn the splendid private mansion adjoining, and while men were shot down and beaten to death before her very eyes, and she became a fugitive in the streets through pools of blood running before her door, wandering with her little children for days and nights together, from the house of one friend, and when it was no longer safe to another, sometimes admitted and sometimes not, as they were "warned" in turns, of intended depredations, it was then she wrote on the blank leaf of her bible, which she always kept at hand for reference, and in this case appending the date,

"In every new distress,  
I'll to His house repair,  
I'll call to mind his wondrous grace,  
And seek deliverance there."

She habitually endeavored to recognize the hand of Providence in the daily affairs of common life. Thus, on one occasion, having left home with her children for a summering at the Delaware Water Gap, and having arrived safely, she wrote promptly to her aged mother, whose affectionate and sleepless solicitude she so well understood and appreciated, "We arrived here very safely and pleasantly. Dear mother! thee sees how much uneasiness we often give ourselves, simply for want of faith. I believe it is best to trust the Lord fully, not by the halves, as thee and I do. I desire to thank Him for our journey, so entirely free from accident, or any unpleasant occurrence; let us for the future trust and love Him more and

For some years, and more repeatedly during her last illness, she requested her husband and her children to read the eighth chapter of Romans. All Christian people love to dwell and feed on verses 1, 28, 32, and the last three; verse eighteen was especially scored with a pencil. Her next favorites were the fourteenth of John, "In my Father's house are many mansions," etc.; and the twenty-third Psalm, "The Lord is my Shepherd." The beginnings of other marked verses will show what impressed her mind most:

"The Lord is my Rock."

"Wait on the Lord."

"Into Thy hand I commit my spirit."

"But I am poor and needy."

"Trust in Him at times."

"Out of the depths have I cried unto Thee, O Lord."

These and many others were marked and dated in her own hand-writing; this last in the month of her mother's death. Her sense of justice towards the poor, and her sympathy for them was such, that if by any means a seamstress or washer-woman, or any other day-worker, left the house without being paid, she was always disturbed and would say, sighingly, "no doubt they needed the money and went away disappointed, after working hard all day, too."

Her exactness in pecuniary transactions was such, that when any of the children would deposit with her any of their little monies for safe-keeping until needed, she never failed to make a memorandum, with day, date and amount, to the fraction of a cent, and to whom it belonged, in a blank book, with pencil attached for that and similar purposes, and all this with a neatness and distinctness which made mistakes impossible from blurs, corrections, or any other cause.

Reader, is it not well to make the Bible our daily companion through life, so that at its close, when all human help is powerless, we may be able to lean upon its beloved words of comfort, and pillow our dying heads on its promises?

